# Finite element analysis of the fracture statistics of self-healing ceramics

**DOI:** 10.1080/14686996.2020.1800368

**Published:** 2020-09-11

**Authors:** Shingo Ozaki, Marika Nakamura, Toshio Osada

**Affiliations:** a Division of System Research, Faculty of Engineering, Yokohama National University, Yokohama, Japan; b Superalloy and High-Temperature Materials Group, Research Center for Structural Materials, National Institute for Materials Science, Ibaraki, Japan

**Keywords:** Self-healing, damage, finite element method, fracture stress, Weibull distribution, 107 Glass and ceramic materials

## Abstract

Self-healing materials have been recognized as a promising type of next-generation materials. Among them, self-healing ceramics play a particularly important role, and understanding them better is necessary. Therefore, in this study, we applied the oxidation kinetics-based constitutive model to finite element analysis of a series of damage-healing processes in self-healing ceramics (alumina/SiC composites). In the finite element analysis, the data on the microstructure distribution, such as relative density, size and aspect ratio of pores, and grain size, were taken as input values and reflected onto the parameters of a continuum damage model using a fracture mechanical model. We then performed a 3-point bending analysis, to consider both the self-healing effect under certain temperature and oxygen partial pressure conditions and scatter of the strength of the ceramics. Our results confirmed that the proposed methodology can reasonably reproduce both strength recovery and damage propagation behavior in self-healing ceramics.

## Graphical abstract

**Figure:**
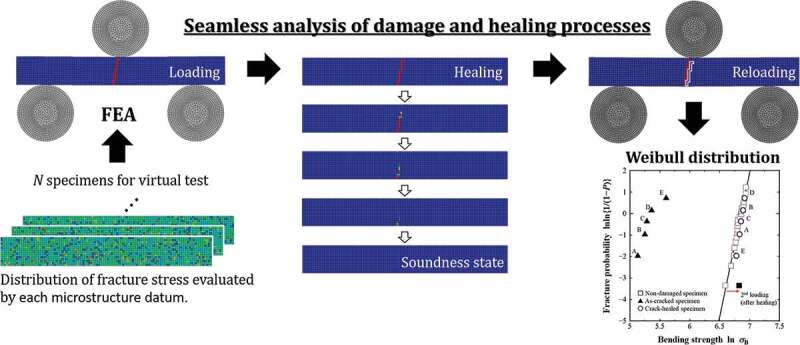

## 1. Introduction

Self-healing materials are considered as an important part of next-generation structural designs. In general, these materials utilize chemical reactions for autonomous repair in service, thus ensuring structural integrity. During the last several decades, numerous studies have been conducted to develop various self-healing materials including polymers, concrete, metals, and their composites [1].

In particular, self-healing ceramics were studied as new functional compounds characterized by low weight and high heat resistance [2–9]. When a micro-crack propagates in self-healing ceramics, rebonding of the crack occurs under high temperature. Specifically, these materials include healing agents, which are non-oxide particles or mediums, and an induced chemical reaction (high-temperature oxidation kinetics) of the healing agent. Thus, the strength of the material autonomously recovers to its initial – or even more robust – state because the crack is rebonded by the oxidation product.

Among various types of crack-healing in ceramics, silicon carbide (SiC) oxidation is quite effective in achieving full strength recovery [10–12]. Thus far, many studies have reported on the crack-healing behavior resulting from the oxidation of alumina/SiC particle composites [13–17]. However, to apply the self-healing materials to various components of machines and structures, a novel numerical simulation method should be developed for evaluating both the damage and the healing processes. Under such circumstances, recent developments have been observed in formulating both damage and healing processes within frameworks of continuum damage mechanics [18–26] and cohesive zone modeling [27].

In particular, the authors have proposed a continuum damage model that incorporates the effects of oxidation kinetics on the strength recovery rate of self-healing ceramics [28,29]. In addition, the proposed model was implemented in the finite element method (FEM) to demonstrate the numerical analysis of alumina/SiC particle composites under various conditions of temperature and oxygen partial pressure.

At the same time, ceramics are brittle materials, and it is known that their stochastic fracture is caused by internal defects, such as pores and flaws [30–35]. Hence, the scatter of fracture strength is observed even in the same lot of test specimens and structural members. Therefore, the evaluation of the fracture strength scatter of bulk bodies, including healed parts – in addition to the fracture strength scatter of virgin, i.e., non-damaged materials – is an essential issue for practical application in self-healing ceramics. For example, in the case of self-healing ceramics, even if the initial crack caused by an internal defect is rebonded, the second crack initiation upon subsequent loading is expected to start from the healed part or defects around it. The fracture strength in such situations depends not only on the degree of strength recovery of the healed part – which depends on time, temperature, and oxygen partial pressure – but also on the spatial distribution characteristics of peripheral defects, in addition to boundary conditions.

In this study, we propose a finite element analysis (FEA) method for fracture statistics of self-healing ceramics by combining a numerical simulation of the damage-healing process with the prediction method of scatter of ceramic strength proposed by the authors [36,37]. Concretely, we conducted the following investigation:

(1) Performing a series of simulations regarding crack initiation, propagation, and healing considering a stochastic distribution of microstructures (relative density, pore, and grain size). Here, the three-point bending tests for non-damaged, as-cracked, and crack-healed specimens were virtually performed.
(2) Analyzing fracture statistics for crack-healed ceramics and discussing the strength scatter before and after healing using the Weibull distribution generated by FEA.

In addition to this examination, a qualitative comparison of the experimental results [15] was performed to demonstrate the effectiveness of the method used for the analyses of fracture statistics of self-healing ceramics. It should be noted that the ceramics we examined were Al_2_O_3_/15 vol% SiC, which were also used in previous studies [15,36,37]. The processing defects on their surfaces were repaired in advance through heat treatment. Thus, it was possible to consider only the fractures that start from internal flaws [8].

The paper is organized as follows: The formulation of the damage-healing constitutive model is described in Section 2, and the fracture mechanics model and the distribution characteristics of microstructure data are explained in Section 3. The numerical models of FEA and the boundary condition are provided in Section 4. Then, the analysis results of FEA and the discussion are described in Section 5. Finally, conclusions and future work are summarized in Section 6.

## 2. Constitutive model

In the isotropic damage and self-healing constitutive model proposed by the authors [28], the damage and healing phenomena are described based on continuum damage mechanics. In this section, we briefly explain how the constitutive model incorporates the evolution laws for self-healing. In the formulation, the damage process is described by the cohesive-force embedded isotropic damage model [36–39], and the self-healing process is prescribed by the evolution laws based on the empirical oxidation kinetics equation [15]. For details on the formulation, see Ozaki et al. [28].

### 2.1 Damage model

The stress–strain relationship based on the typical isotropic damage model is given as follows:

(1) σ=(1−D)c:ε

where **σ**, **ε**, and **c** are the Cauchy stress tensor, small strain tensor, and fourth-order elastic coefficient tensor, respectively. *D* (0≤D≤1)is the damage variable, and *D* = 0 and *D* = 1 correspond to the non-damaged state and perfectly damaged state, respectively. To describe the dependency of damage history, the damage variable *D* is formulated as a function of the maximum value of equivalent strain *κ* in the loading history, and is given as below:

(2) $$D(\kappa )=1-\frac{\kappa_{0}}{\kappa }\exp\left\{-\frac{\sigma_{t}h_{e}}{G_{f}}(\kappa -\kappa_{0})\right\}$$

Here, *κ* can be considered a state variable. $\kappa_{0}$ is the equivalent strain at damage initiation, *h_e_* is the characteristic length (which corresponds to the length of the element in FEA), *σ_t_* is the fracture stress, and *G_f_* is the fracture energy.

### 2.2 Evolution laws for state variables

In alumina/SiC composites, the self-healing behavior is achieved by the passive oxidation of SiC. Based on the empirical oxidation-kinetics [15], the average crack-healing rate, *v_h_*, for complete strength recovery is given by

(3) $$v_{h}=A_{h}\exp\left(\frac{-Q_{h}}{RT_{h}}\right)(a_{O2}{)}^{3n/2}$$

Here, *Q_h_* [kJ/mol] is the activation energy for crack healing, *A_h_* [s^–1^] is the frequency factor, *R* [J/mol] is the gas constant, and *T_h_* [K] is the healing temperature. Furthermore, *n* is the temperature-independent reaction order of O_2_ and $a_{O_{2}}=P_{O_{2}}^{\;}/P_{O_{2}}^{o}$ is the oxygen pressure. Here, $P_{O2}$ is the partial pressure of oxygen and $P_{O2}^{o}$ is the standard pressure of 0.1 MPa.

To incorporate the self-healing behavior into the damage model described above, it was assumed that the damage variable evolves from the state of D≠0 towards *D* = 0 through the self-healing, depending on the temperature and oxygen partial pressure. In other words, the damage history disappears with self-healing, and the state variable *κ* evolves to the soundness state. Therefore, it was also assumed that the state variable *κ* additively decomposed into the equivalent strain (damage) part $\kappa_{\varepsilon }$ and the self-healing part $-\kappa_{h}$ as follows:

(4) $$\kappa =\kappa_{\varepsilon }-\kappa_{h}$$

where the evolution of the damaged part is given by the following equation.

(5) $$\left.\begin{array}{cc} {\overset{\cdot }{\kappa }}_{\varepsilon }=\langle \overset{\cdot }{\varepsilon_{eq}}\rangle & if\;\kappa_{\varepsilon }=\varepsilon_{eq},\\ {\overset{\cdot }{\kappa }}_{\varepsilon }=0\; & \;\;if\;\;\;\kappa_{\varepsilon }>\varepsilon_{eq}. \end{array}\right\}$$

Here, <> stands for McAuley’s brackets. We adopted the following modified von-Mises equivalent strain $\varepsilon_{eq}$, which is a scalar value, for the evolution of damage:

(6) $$\varepsilon_{eq}=\frac{k-1}{2k(1-2\nu )}I_{1}+\frac{1}{2k}\sqrt{{\left(\frac{k-1}{1-2\nu }I_{1}\right)}^{2}+\frac{12k}{{\left(1+\nu \right)}^{2}}J_{2}}$$

where ν is Poisson’s ratio, *k* is the ratio between the tensile and compressive strengths, *I*_1_ is the first invariant of the strain tensor, and *J*_2_ is the second invariant of the deviatoric strain tensor.

Conversely, the self-healing part $\kappa_{h}$ is assumed to be a monotonic increasing function, which describes the process $\kappa \to \kappa_{0}$. Thus, we adopted the following function for the evolution rule of $\kappa_{h}$:

(7) $${\overset{\cdot }{\kappa }}_{h}=\xi_{1}\frac{w_{exp}}{(\kappa_{\varepsilon }-\kappa_{0})h_{e}}v_{h}\left(\kappa -\kappa_{0}\right)\;\;\;\;for\;\;\;\kappa_{\varepsilon }>\kappa_{0}$$

where $\xi_{1}\;\;(>0)$ is the parameter that influences the self-healing rate, and *w_exp_* is the crack mouth opening displacement (maximum crack-opening width) in the experiment [15].

Furthermore, to describe the dependence of the strain at damage initiation after healing on the degree of self-healing, the maximum equivalent strain $\overset{ˉ}{\kappa }$ received in the past must be re-evaluated. For the self-healing state, we assumed that the maximum equivalent strain $\overset{ˉ}{\kappa }=\max\{\kappa_{\varepsilon }\}\geq \kappa_{0}$ gradually approaches $\kappa_{s}$, and the evolution rule for the maximum equivalent strain can be expressed as follows:

(8) $$\overset{\cdot }{\bar{\kappa }}=-\xi_{2}\frac{w_{exp}}{(\kappa_{\varepsilon }-\kappa_{0})h_{e}}v_{h}\left(\overset{ˉ}{\kappa }-\kappa_{s}\right)\;\;\;\;for\;\;\;\kappa_{\varepsilon }>\kappa_{0}$$

In the reloading state after healing, damage does not occur until $\kappa \geq \overset{ˉ}{\kappa }$. Here, $\xi_{2}\;\;(\geq 0)$ is the parameter affecting the self-healing rate. It should be noted that when $\kappa_{s}>\kappa_{0}$, the fracture strength of the healed part becomes higher compared with the non-damaged part. This phenomenon is caused, not only by the strengthening of the healed part but also by the scatter of the strength due to internal pores. Therefore, $\kappa_{s}>\kappa_{0}$ means that the internal pore – which was an origin of initial crack – is also filled by the oxide, and then, the fracture strength after healing depends on spatial distribution characteristics of peripheral pores. Thus, $\kappa_{s}$ should be defined by referring to microstructure data after complete healing [16].

The judgment of damage and the loading criterion are described by

(9) $$\left.\begin{array}{c} if\;\;\;\kappa <\kappa_{0}\;\to D=0\;\;\;\;for\;\;undamaged,\\ if\;\;\;\kappa <\overset{ˉ}{\kappa }\;\to D=D(\kappa )\;\;\;\;for\;\;\kappa \geq \kappa_{0},\;\;\;\\ if\;\;\;\kappa \geq \overset{ˉ}{\kappa }\;\to D=D(\kappa )\;\;\;\;for\;\;\kappa =\overset{ˉ}{\kappa }.\;\;\;\;\; \end{array}\right\}$$

(10) $$\left.\begin{array}{c} \left\langle {\overset{\cdot }{\varepsilon }}_{eq}\right\rangle >0:\;\;\;\;\;\;\;loading,\;\;\;\;\;\;\\ \left\langle {\overset{\cdot }{\varepsilon }}_{eq}\right\rangle =0:\;\;\;\;\;\;unloading,\\ \left\langle {\overset{\cdot }{\kappa }}_{h}\right\rangle >0:\;\;\;\;\;\;\;\;healing.\;\;\;\;\;\;\; \end{array}\right\}$$

Note that the initial value of $\overset{ˉ}{\kappa }$ for non-damaged state corresponds to $\kappa_{0}$.

### 2.3 Response characteristics of the damage-healing constitutive model

**Figure 1.**
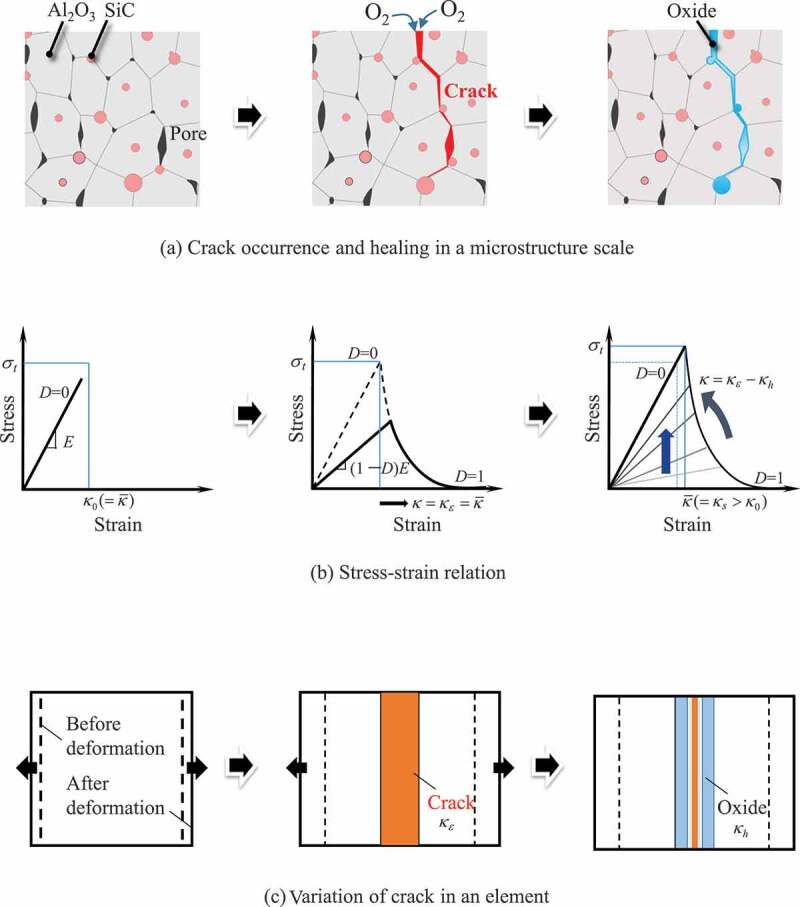
Schematic response characteristics of damage-healing processes by the damage-healing constitutive model under uniaxial tensile conditions: (a) crack occurrence and healing on a microstructural scale; (b) stress–strain relation; and (c) variation of cracks in an element with an embedded cohesive zone relationship.

Figure 1 shows the schematic response characteristics of damage-healing processes by the present constitutive model under uniaxial tensile conditions. Here, Figure 1(a) presents crack occurrence and healing on a microstructure scale, Figure 1(b) corresponds to the stress–strain relation, and Figure 1(c) shows the variation of cracks in an element in which the cohesive zone relationship is embedded.

As shown in the figure, the constitutive model shows elastic behavior until the equivalent strain reaches $\kappa_{0}\;\;(=\overset{ˉ}{\kappa })$, and transitions to D≠0 state with crack initiation; then, stiffness decreases according to the magnitude of *D*. It should be noted that the equivalent strain at damage initiation, $\kappa_{0}$, of each element is evaluated by the scatter of the fracture stress, *σ_t_*, due to microstructure information and the elastic relation assumed to be in uniaxial relation (see next section). After unloading, the oxidation reaction starts with the initiation of cracking and the crack is filled with oxides. At the same time, the equivalent strain κ evolves depending on the temperature and oxygen partial pressure, and the damage variable recovers to *D* = 0. In addition, the maximum equivalent strain $\overset{ˉ}{\kappa }$ evolves in response to the disappearance of the pore that is the origin of the initial crack, as the cracked part is sufficiently healed. Therefore, in subsequent loading, the fracture will occur starting from the next largest pore in the periphery of the healed part.

## 3. Stochastic distribution of fracture parameters

The crack initiation in self-healing ceramics is caused by inherent defects. Therefore, such fracture behavior must be reflected in the FEA. The most physically reasonable fracture mechanics model of ceramics is a combination of a stress concentration part (pore) and an initial crack [40–45]. In the FEA methodology previously proposed by the authors [36,37], the damage model parameters are evaluated automatically, based on the distribution of information regarding microstructure (relative density, size and aspect ratio of pores, and grain size), by means of a fracture mechanics model. Therefore, the microscopic structure information obtained through image observation becomes the input value for the FEA.

In this section, the fracture mechanics model and the probability density function of each microscopic structure data are outlined. For details of the formulation, see Ozaki et al. [36].

### 3.1 Fracture mechanics model

Relative density, size and aspect ratio of pores, and grain size are stochastically distributed in a ceramic body because of sintering. Therefore, the parameters of the damage model for each element (or integration point) differ depending on these values. It should be noted that, in this study, we focus only on mode I cracks caused by tension.

The mechanical properties of ceramics that contain many pores are characterized by porosity. According to Flinn et al. [45], the relationship between porosity *P* (or relative density ρ) and the apparent Young’s modulus *E* is expressed by the following equation:

(11) $$E=E_{100}{\left(1-\frac{P}{P_{1}}\right)}^{\alpha },\;\;\;\;P=1-\rho$$

where *E*_100_ is the Young’s modulus for a fully dense sample, and $P_{1}$ and exponent *α* are the fitting parameters. Referring to Flinn et al. [45], we set $P_{1}=0.45$ and α=1.15.

**Figure 2.**
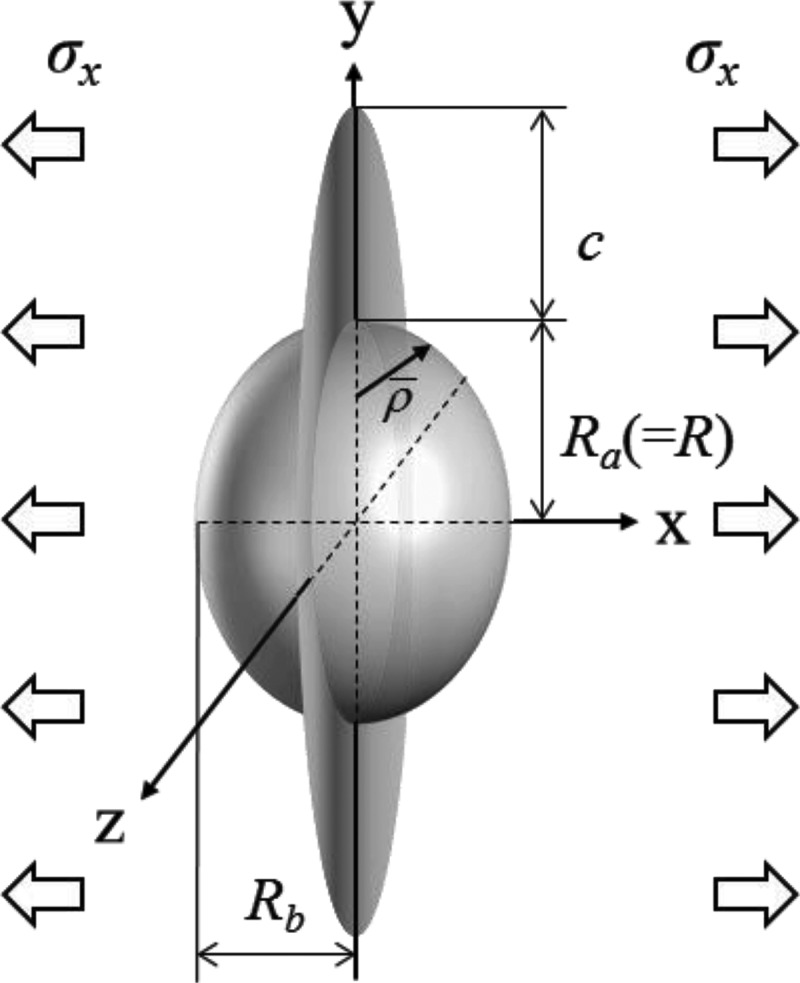
Fracture mechanics model based on an ellipsoidal (spheroidal) pore [35].

In this study, the fracture stress *σ_t_* was estimated based on the linear fracture mechanics, as follows:

(12) $$\sigma_{t}=\frac{K_{IC}}{F}\frac{1}{\sqrt{\pi c}}$$

where *K*_IC_ is the fracture toughness, and *c* is the initial crack length. *F* is the geometric factor obtained from the shape of an ellipsoidal (spheroidal) pore and the initial crack length, as shown in Figure 2. Here, *R_a_* = *R* is the major axis radius, and *R_b_* is the minor axis radius. Thus, the aspect ratio *A* of the ellipsoidal pores is defined as *R_b_*/*R*.

The geometric factor for a circumferential crack emanating from an ellipsoidal pore under tension (Figure 2) is summarized as follows:

(13) $$F=\left\{\begin{array}{c} F_{1}\;\;\;\;\;\;\;\;\;\;\;\;\;\;\;\;\;\;\;\;\;\;\;\;\;\;\;\;\;\;\;for\;\;\;\;c/\overset{ˉ}{\rho }\leq 1,\\ \;\;\;\;max(F_{1},\;\;F_{2})\;\;\;\;\;\;for\;\;\;\;c/\overset{ˉ}{\rho }>1. \end{array}\right.$$

Here,

(14) $$\begin{array}{rl} F_{1}= & 1.125K_{t}\left[\frac{1}{3}+\frac{1}{6}\left\{\frac{1}{{\left(1+\lambda \right)}^{2}}+\frac{3}{{\left(1+\lambda \right)}^{4}}\right\}\right]\\ & \left(1+0.2238\lambda -0.1643\lambda^{2}\right) \end{array}$$

(15) $$F_{2}=\frac{2}{\pi }\sqrt{\frac{R+c}{c}}$$

where $\overset{ˉ}{\rho }$ is the notch root radius, and $\lambda =c/\overset{ˉ}{\rho }$. In this study, we assumed that the initial crack length *c* corresponds to grain size. Concrete equations, such as stress concentration factor *K_t_*, were provided in Ozaki et al. [36].

The fracture energy used in the damage model is given as follows:

(16) $$G_{f}=\frac{K_{IC}^{2}}{E}\left(1-\nu^{2}\right)$$

In addition, the equivalent strain at damage initiation, $\kappa_{0}$, was set by assuming an uniaxial tensile fracture as follows:

(17) $$\kappa_{0}=\sigma_{t}\frac{\left(1+\nu )(1-2\nu \right)}{E(1-\nu )}$$

### 3.2 Probability density functions for microstructure data

In the present FEA methodology, different relative densities, sizes and aspect ratio of pores, and grain sizes are automatically set for each element (or integration point) by using the probability density function and random numbers. Then, the parameters are evaluated by Eqs. (11), (12), (16), and (17). Therefore, the average value and standard deviation of microstructure data are used as input values. The probability density functions of each microstructure datum are described below.

#### Relative density ρ

3.2.1

The distributions of relative density were defined using probability density functions of the half-normal distribution with a maximum value $\mu_{\rho }$ and a standard deviation$\sigma_{\rho }$.

#### 3.2.2 Pore size R

As clarified, that the larger the pores in the ceramics, the lower the probability of their existence. The distribution of pore size was defined by the probability density function of the half-normal distribution with a minimum value $\mu_{R}$ and a standard deviation $\sigma_{R}$. Although many small pores exist within the size of one element, we pay attention only to the largest pore that can be the origin of crack in each element. Therefore, the minimum value corresponds to the maximum pore size that would exist in the volume per element. At the same time, the standard deviation prescribes the maximum pore size that may exist in the total volume of the analyzed body.

It was pointed out that the pore size distribution characteristics of ceramics often follow a power law. It should be noted that the present method can also use a power law distribution as a probability density function of pore size [37].

#### 3.2.3 Aspect ratio of pore A

The aspect ratio of pores was defined by the normal distribution, with a mean value $\mu_{A}$ and a standard deviation $\sigma_{A}$, based on the cross-sectional image observation results of Al_2_O_3_/15 vol% SiC, which was produced by pressure sintering.

#### 3.2.4 Grain size (length of initial crack) c

Grain growth during sintering is explained by Ostwald ripening. Therefore, the distribution characteristics of the grain size were modeled by the lognormal distribution with a mean value $\exp\left(\mu_{c}+\sigma_{c}^{2}/2\right)$ and a standard deviation $\sqrt{e^{2\mu_{c}+\sigma_{c}^{2}}(e^{\sigma_{c}^{2}}-1)}$.

From the above, the parameters required for the constitutive model are obtained by providing the microstructure dataset (ρ, *R, A*, and *c*) and the basic material properties ($E_{100}$, $P_{1}$, *α*, ν, $K_{IC}$, and *k*), in addition to the self-healing parameters.

## 4. Finite element model and analysis conditions

In the FEA, we used a commercial software package LS-DYNA and its related user subroutine [46]. For the analysis of the damage process, the dynamic explicit method based on the central difference method was adopted, whereas, for the analysis of the self-healing process, the dynamic implicit method based on the time discretization of the Newmark *β* method was adopted. The restart function of LS–DYNA was utilized for connecting each process, consisting of the loading, healing, and reloading stages, because the timescales of the loading/unloading stages were very different compared with the healing one. The specification of finite element (FE) models and analysis condition are described below.

### 4.1 FE model and boundary conditions

**Figure 3.**
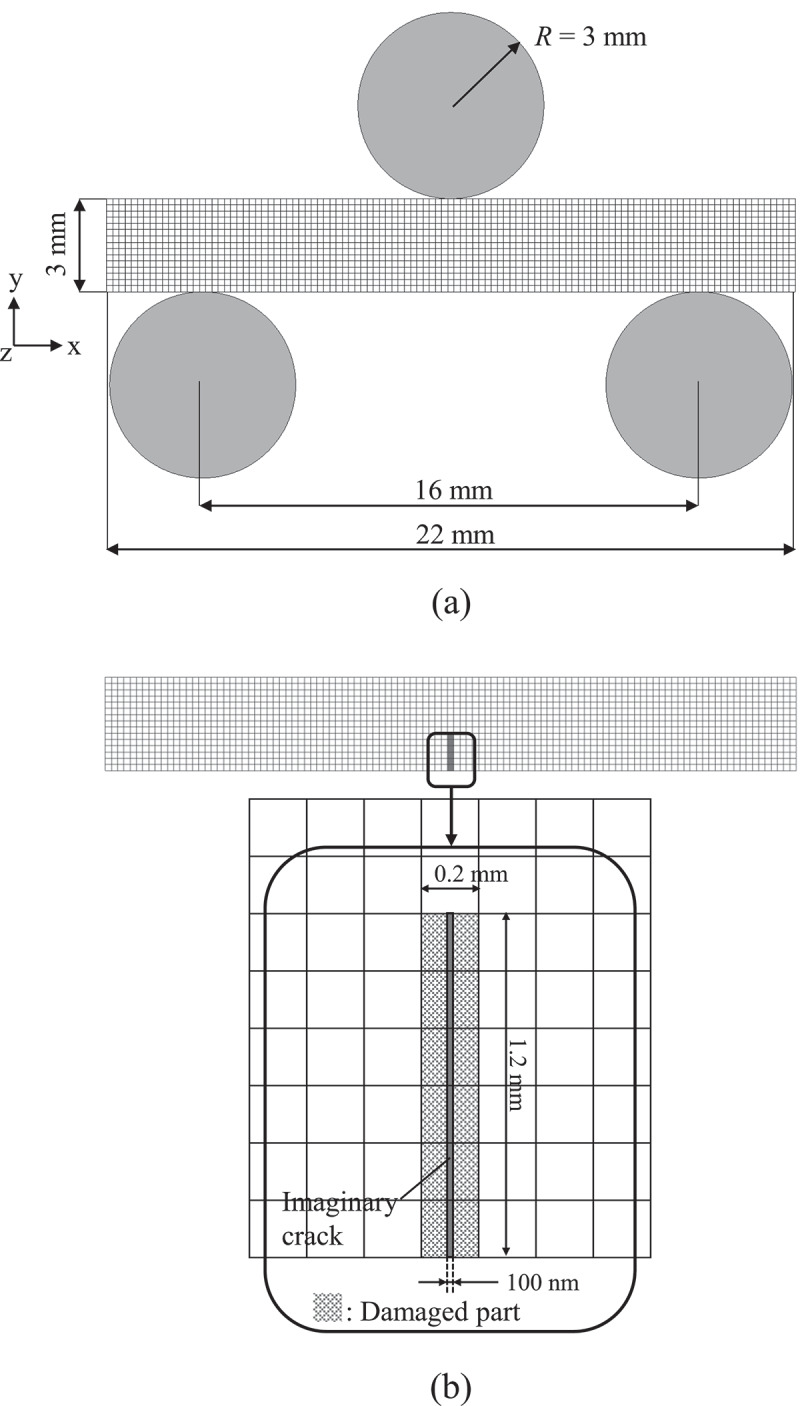
FE models of the three-point bending test and healing: (a) non-damaged specimen; (b) as-cracked specimen and the details of initial damaged part.
(2) As-cracked specimen (Figure 3(b))
(3) Crack-healed specimen, which corresponds to a healed specimen of as-cracked specimen.

In this study, the 3-point bending test was adopted as the analysis target with reference to the previous experiments [8,15]. However, to simplify the examination of the effects of the strength variation on the self-healing, the width of the specimen (z-direction) was set as one element, assuming the plane strain conditions. The following three types of test specimen models were used for the analysis:

(1) Non-damaged specimen (Figure 3(a))

Here, the length of the pre-crack in the as-cracked specimen was 1.2 mm, referring to Ono et al. [8].

**Figure 4.**
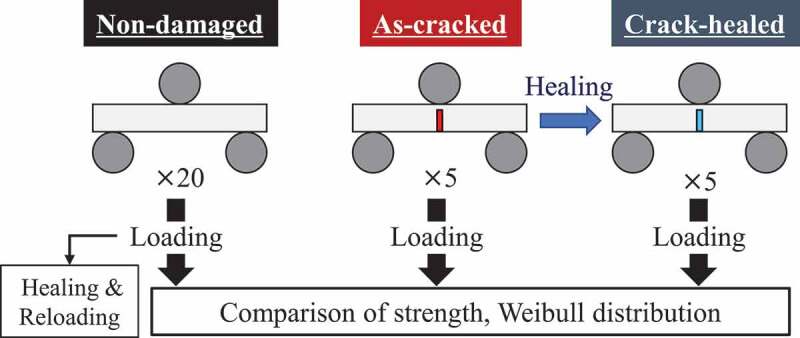
Simulation flow.

To create the Weibull distribution, *N* = 20 non-damaged specimens and *N* = 5 as-cracked specimens were analyzed. Meanwhile, to confirm the strength recovery by self-healing, *N* = 5 crack-healed specimens were also analyzed. The simulation flow is summarized in Figure 4.

The jigs of the 3-point bending test were set as rigid bodies. In bending test calculations, a constant forced velocity (constant crosshead speed of 5 mm/s) in the vertical direction was imposed on the upper jig, while the displacements of the lower jigs were fixed. A friction coefficient of 0.3 was set at the contact boundary between the specimen and jigs. Meanwhile, in the self-healing process, the jigs were ignored, and the displacement condition of specimens was completely fixed. Then, the self-healing under prescribed constant temperature and oxygen partial pressure conditions was analyzed. In addition, strength evaluation of crack-healed specimens used the 3-point bending jigs under the same boundary condition. It should be noted that stress, strain, and state variables in each element were inherited between damage and healing analyses, and vice versa.

### 4.2 Input parameters

Tables 1 and 2 show the mechanical and self-healing parameters of the Al_2_O_3_/15 vol% SiC, respectively. Table 3 shows the distribution characteristics of the microstructure. These values are determined by referring to the previous studies [28,29,36]. Regarding the atmospheric conditions for the self-healing process, constant values of healing temperature *T_h_* and oxygen partial pressure $a_{O_{2}}$ were set.

**Table 1.** Mechanical parameters of Al_2_O_3_/15 vol% SiC.

*E* [GPa]	*ν*	*K*_IC_ [MPa mm^0.5^]	*k*
398	0.21	120	11.3

**Table 2.** Parameters for self-healing of Al_2_O_3_/15 vol% SiC.

*A_h_* [s^−1^]	*Q_h_* [J/mol]	*R* [J/(mol/K)]	*n*	*w_exp_* [μm]
1.04 × 10^4^	3.87 × 10^5^	8.31	0.836	0.1
$\xi_{1},\;\;\;\xi_{2}$	*T_h_* [K]	$a_{O_{2}}$	$\kappa_{s}$	
5.0	1623	0.21	4750 × 10^−6^

**Table 3.** Microstructure distribution characteristics.

Relative density		Pore size [μm]		Aspect ratio		Grain size [μm]	
*μ_ρ_*	*σ_ρ_*	*μ_R_*	*σ_R_*	*μ_A_*	*σ_A_*	*μ_c_*	*σ_c_*
0.99	0.001	0.437	3.18	1.0	0.5	0.6	0.1

The pre-crack part shown in Figure 3(b) was considered as an initial damaged part. The value of the initial damage was set to *D* = 0.93, which corresponds to $\kappa =\kappa_{\varepsilon }=\overset{ˉ}{\kappa }=500\times 10^{-6}$. Here, the equivalent strain at damage initiation of initial damaged part, $\kappa_{0}$, was determined by the scale parameter of Weibull distribution obtained by the experiment [15] and Eq. (17).

As the microstructure data before and after self-healing differ, the material properties also differ. The mechanical parameters of the healed part were determined by referring to the microstructure observation results [16]. Specifically, the relative density was set to *ρ *= 0.99, which was the same as before healing. The pore size after healing was *R_h_ *= 0.29 [μm]. The aspect ratio was *A_h_ *= 0.6. Although the composition of the healed part is mainly SiO_2_, we used the mean value of the grain size before healing, i.e., *c_h_ *= 3.08 [μm]. The fracture stress *σ_t_* and fracture energy *G_f_* were calculated from the determined microstructure data using Eqs. (12) and (16), where the fracture toughness, *K*_IC_, shown in Table 1, was adopted. The strain at damage initiation after complete healing, $\kappa_{s}(>\kappa_{0})$, was determined from the fracture stress (*σ_t_* = 1842 [MPa]) and Eq. (17). Other mechanical parameters are the same as Table 1.

## 5. FEA results and discussion

### 5.1 Results from non-damaged specimens

**Figure 5.**
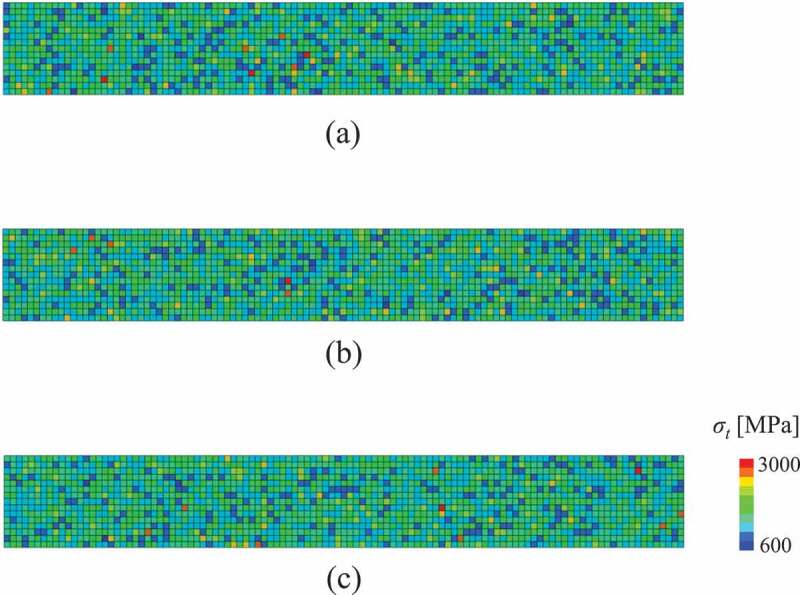
Fracture stress distribution in the three specimens, where the colors in the contour map represent the elemental values. Here, specimens (a), (b), and (c) correspond to specimens of minimum, median, and maximum bending strengths, respectively.

**Figure 6.**
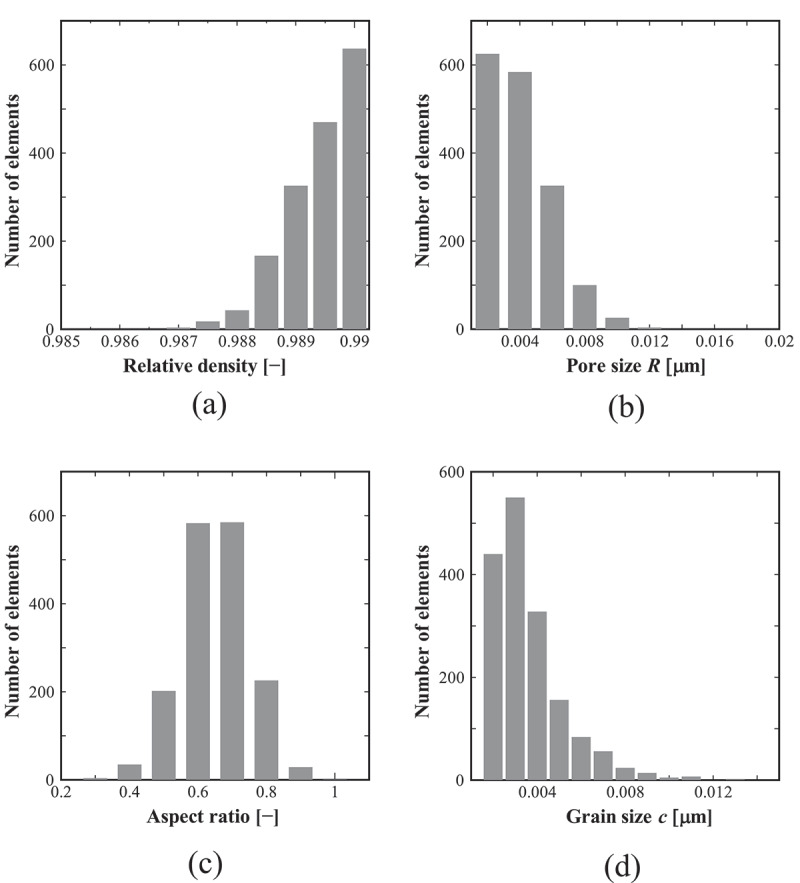
Histogram of the microstructure data of the elements obtained by one specimen: (a) relative density; (b) pore size (major radius); (c) aspect ratio of the pore; and (d) grain size. The vertical axis of the graphs represents the number of elements.

First, we describe the analysis results of non-damaged specimens. Figure 5 shows examples of the contour map of the fracture stress in three specimens. Here, specimens (a), (b), and (c) correspond to the bending strengths of the minimum, the median, and the maximum value among *N* = 20 specimens, respectively. Further, Figure 6 shows histograms of the relative density, pore size, aspect ratio, and grain size of elements obtained from one specimen arbitrary extracted. Reflecting the probability density function of the microstructure distribution shown in Table 3, it can be confirmed that they are dispersed within the specimens. In addition, the results in Figure 5 can confirm that the distribution of fracture stress differs for each specimen by using random numbers.

**Figure 7.**
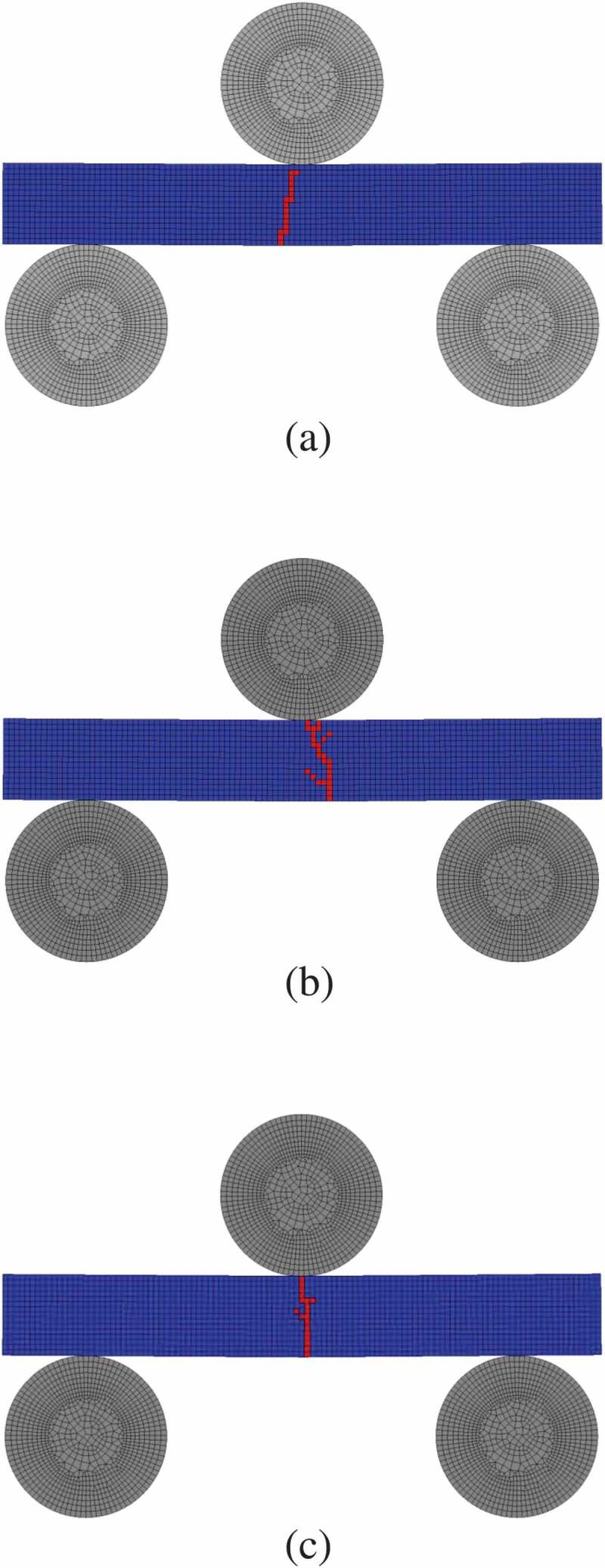
The damage variable distribution in the specimens corresponding to that in Fig. 5. The contour map shows the result of damage propagation in specimens under the three-point bending test. The blue elements represent the state without damage, whereas the red elements represent the damaged state.

**Figure 8.**
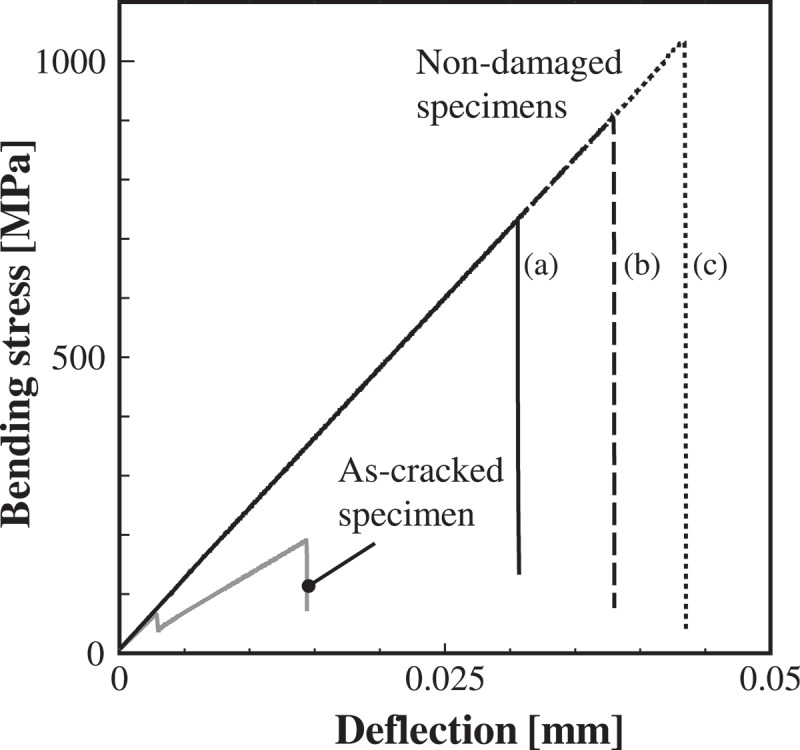
Relationship between the bending stress and deflection obtained by three specimens shown in Fig. 5. The graph includes the result of the as-cracked specimen.

Figure 7 shows the results of the 3-point bending analysis of three specimens shown in Figure 5. Here, the figure depicts the contour map of the damage variable. The blue elements represent the state without damage (*D* = 0), whereas the red elements represent the damaged state (*D* ≈ 1). In addition, Figure 8 shows the relationships between the bending stress and deflection of each specimen. From the figures, it can be observed that the bending stress increases linearly with deflection, the damage progresses when a certain element around the bottom center of specimen reaches the fracture stress, and then the bending stress sharply decreases. Hence, the present FEA model can represent a typical brittle fracture behavior of ceramics. Furthermore, when considering the stochastic distribution of the microstructure, as the damage starts from the weaker element around the bottom center that receives a relatively large tensile stress, the bending strength varies depending on the distribution characteristics of the microstructure. In other words, the path of damage progress and bending strength differ for each specimen.

**Figure 9.**
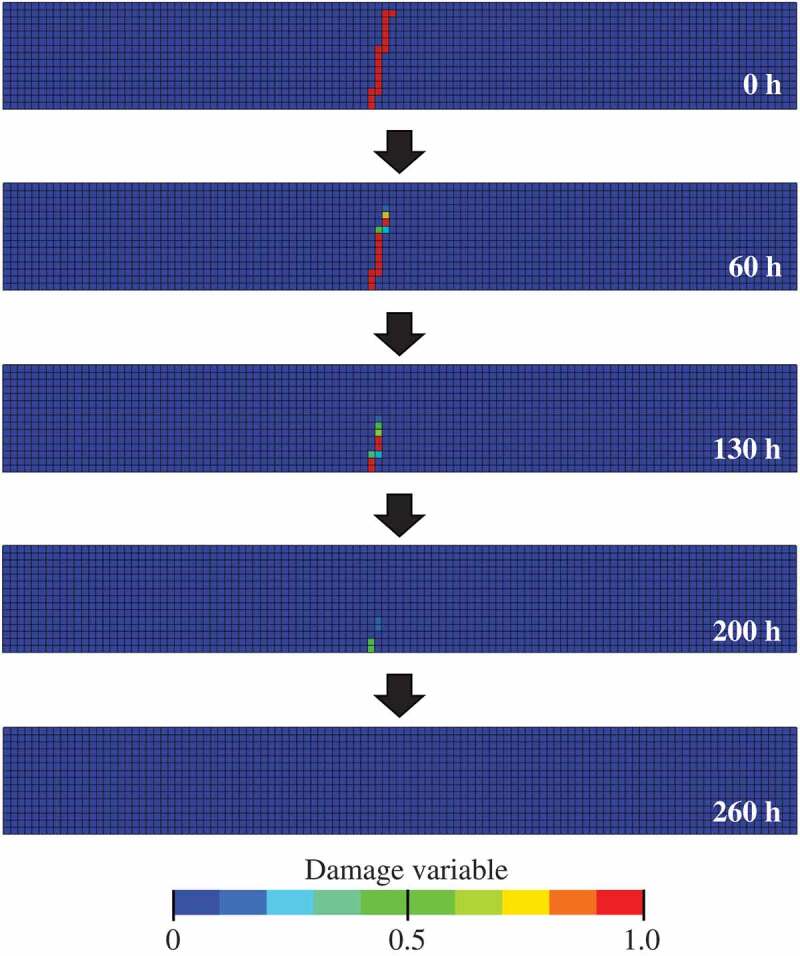
Time series snapshots of the distribution of damage variable *D* in the specimen of Fig. 5(a) during the healing stage under a prescribed condition after the unloading (temperature *T_h_* = 1623 [K] and oxygen partial pressure $a_{O_{2}}=0.21$).

Figure 9 shows a time series variation of snapshots of the damage variable distribution in the specimen shown in Figure 5(a) during healing stage, under prescribed conditions after the unloading. Here, the loading was removed just when the bending stress reached the peak value. It can be observed that the damaged region has recovered to a soundness state (*D* = 0) within the elapsed time. Further, reflecting on Eq. (7), the healing of the damage is attained from the region where the crack-opening width is small.

In this FEA example, the damage introduced by the 3-point bending test was healed and the crack-opening width was larger than that of the crack introduced by the ordinary Vickers indenter [15]. Therefore, the healing time is very long. Note that the example is intended to demonstrate FEA for a series of damage-healing processes.

**Figure 10.**
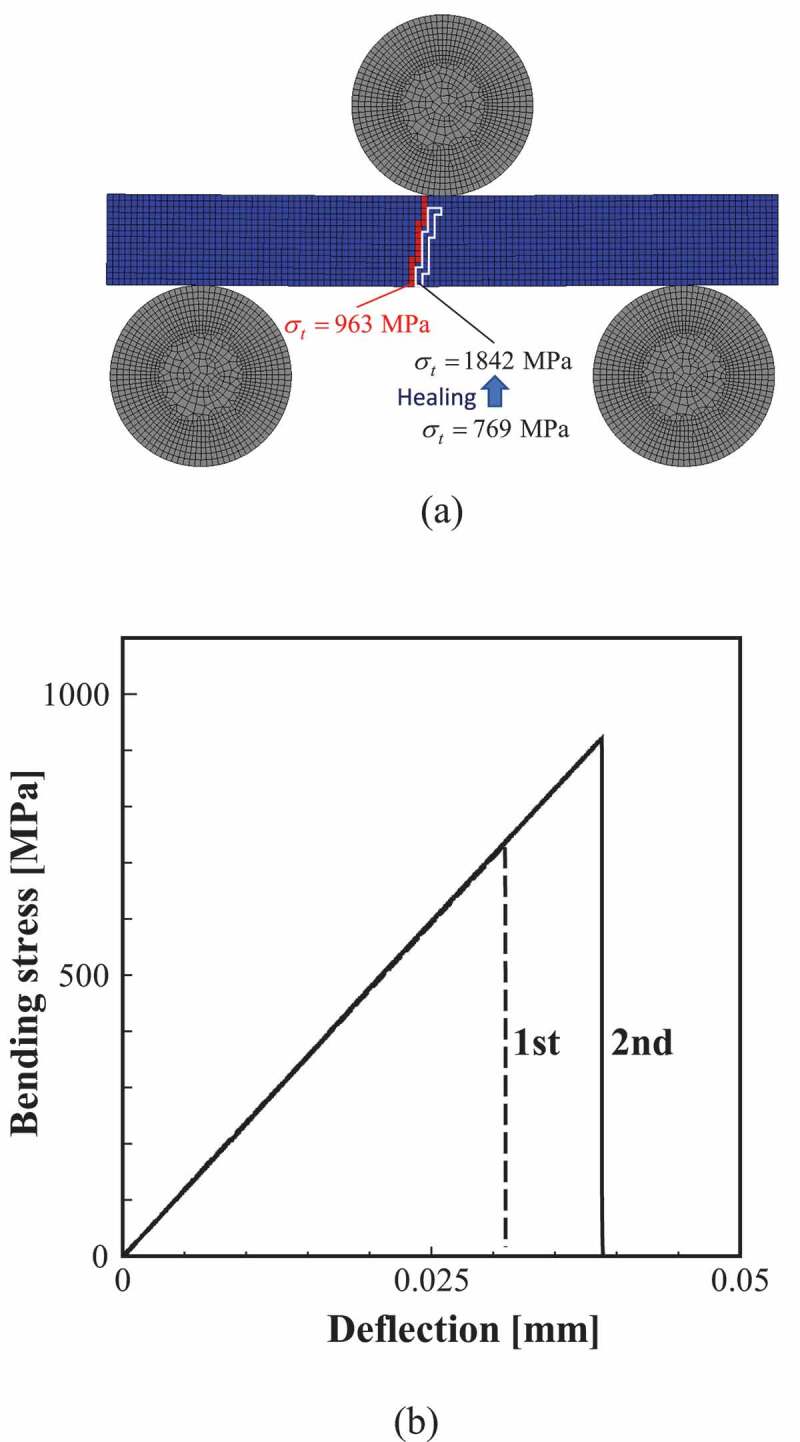
FEA results of reloading for completely healed specimen shown in Fig. 9: (a) the contour map of the damage variable: (b) bending stress–deflection relationship. The white frame in the contour map depicts the path of damage progression in the first loading.

Figure 10 shows the FEA results when the specimen after healing (Figure 9) was reloaded. Here, Figure 10(a) and 10(b) shows the contour map of the damage variable and the bending stress–deflection relationship, respectively. It can be observed that the damage progresses to avoid the previously healed part when reloading. The white frame in Figure 10(a) corresponds to the healed part.

Focusing on the fracture stress of each element, the fracture stress of the element at the damage initiation on the bottom surface of the non-damaged specimen was 769 MPa, whereas the fracture stress increased to 1842 MPa because of the sufficient healing. Therefore, it was confirmed that the fracture origin moved to the next weakest element (963 MPa) around the bottom center where the bending stress was large. As a result, the bending strength in Figure 10(b) also increases.

Bearing in mind the above-mentioned, the present FEA methodology can evaluate the self-healing behavior in conjunction with the simulation of the fracture strength scatter caused by the stochastic distribution of microstructure.

### 5.2 Results from as-cracked and crack-healed specimens

In this section, we describe the analysis results of as-cracked and crack-healed specimens.

**Figure 11.**
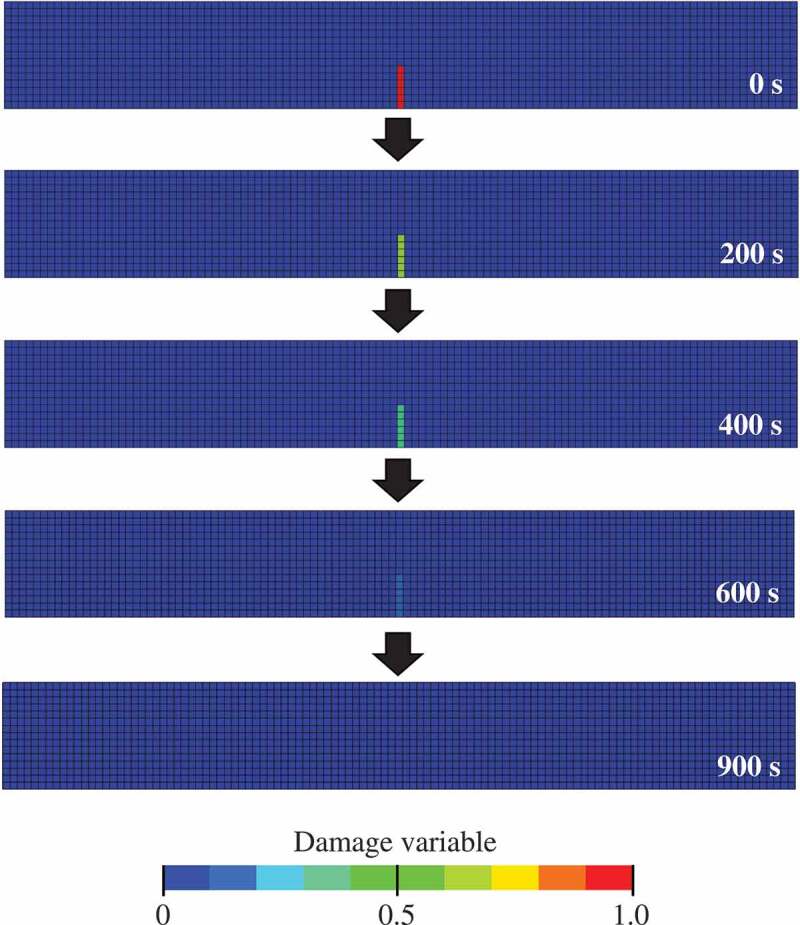
Time series snapshots of the healing process of the initial damaged part in an as-cracked specimen. The blue elements represent the state without damage, whereas the red elements represent the damaged state.

Figure 11 shows time series variation of snapshots of the healing process of the initial damaged part in an as-cracked specimen. Here, the figure depicts the contour map of the damage variable. The blue elements represent the state without damage (*D* = 0), whereas the red elements represent the damaged state (*D* ≈ 1). As in the case of Figure 9, it can be confirmed that the initial damaged part has healed within the elapsed time and changed to a soundness state. The time required for complete healing is approximately 900 s. As the crack size in the initial damage part is based on experiments [8,15], it can be confirmed that the recovery is achieved on the same time scale as the experiment [15].

**Figure 12.**
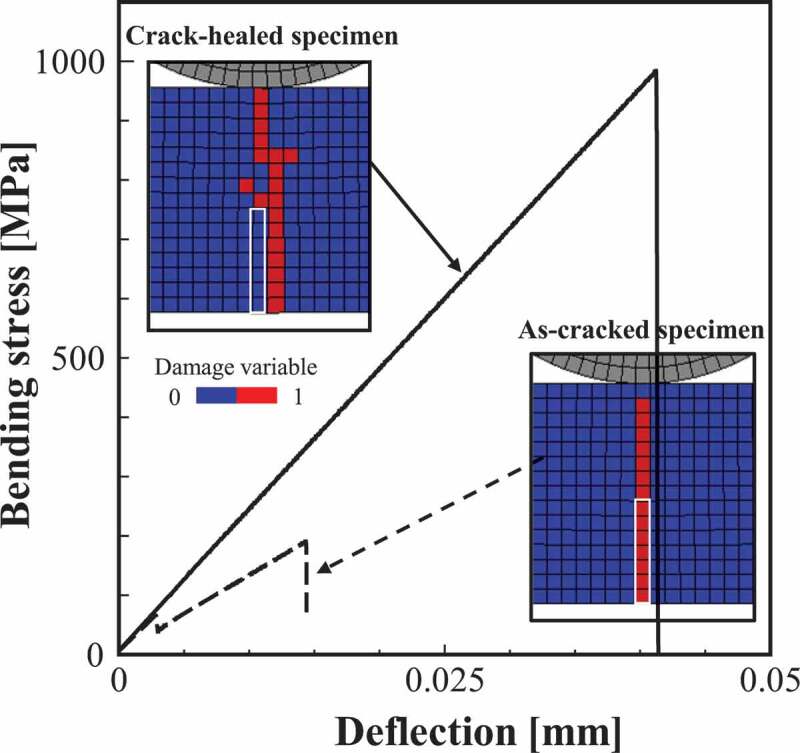
Relationships between bending stress and deflection for as-cracked and crack-healed specimens. The figure also depicts the contour map of the damage variable. The white frame in the contour map depicts the initial damaged part.

Next, the change in bending strength of the as-cracked specimen before and after healing is examined. Figure 12 shows the results of the 3-point bending test for as-cracked and crack-healed specimens. As demonstrated by the bending stress–deflection relationship, although both specimens exhibit brittle fracture, the stiffness and strength of the cracked-healed specimen is clearly larger. The figure also shows the progress of the damage. In the as-cracked specimen, the damage propagates straight from the notch (initial damaged part) because of the stress concentration effect. Contrary to this, in the crack-healed specimen, the damage occurs around the pre-cracked part. As these results confirm, the fracture strength of the initial damaged part is recovered by sufficient healing, and the origin of damage initiation moves to the elements with relatively small fracture stress (elements with large pores or grain size) around it. The bending stress–deflection relationships in Figure 8 also show the results of the as-cracked specimen. As shown in the figure, the as-cracked specimen has lower strength and stiffness than the non-damaged specimens. It should be noted that the crack-healed specimen shown in Figure 12 exhibits a similar bending stress–deflection relationship to that of non-damaged specimens.

Furthermore, to confirm these tendencies of strength recovery and scatter, Weibull distributions were created using the bending strength (peak value of bending stress) obtained from the FEA.

**Figure 13.**
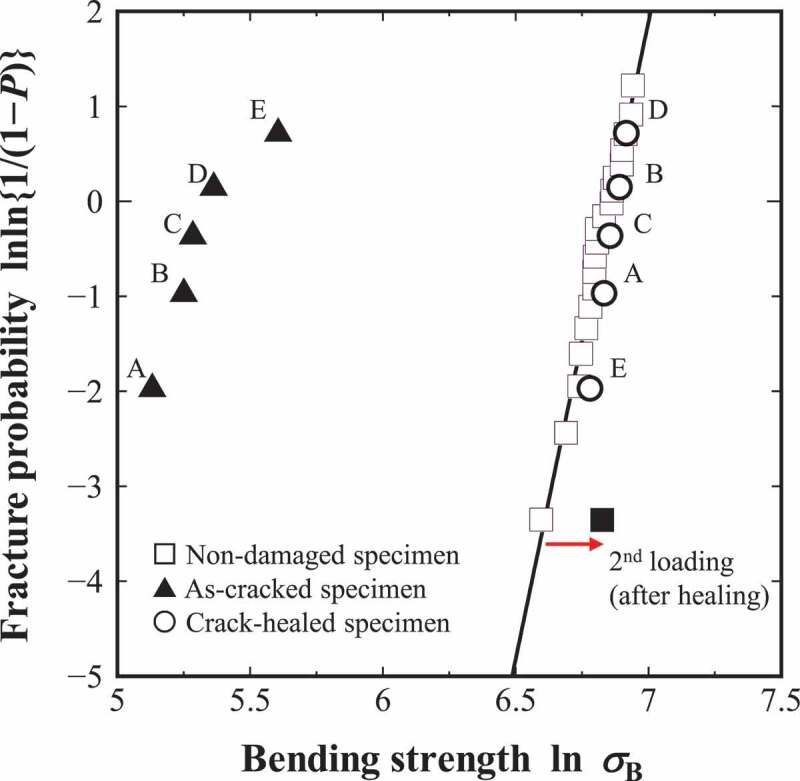
Weibull distributions obtained by the FEA of non-damaged, as-cracked, and crack-healed specimens. The calculated combinations of the Weibull modulus *m* and the scale parameter *β* [MPa] are as follows: non-damaged specimen (13.4, 983 [MPa]), as-cracked specimen (4.5, 225 [MPa]), and crack-healed specimen (19.5, 975 [MPa]).

Figure 13 shows the Weibull distributions of the non-damaged specimen (*N* = 20), as-cracked specimen (*N* = 5), and crack-healed specimen (*N* = 5), where the median rank method was adopted. In the figure, A-E shows the number of the test pieces, which are common between the as-cracked and crack-healed specimens. As shown in the figure, when complete healing is achieved, the results of the crack-healed specimen overlap the Weibull distribution of the non-damaged specimens. In other words, the scatter of strength in the crack-healed specimen is almost the same as that in the non-damaged specimen, and the Weibull modulus *m* and the scale parameter *β* show similar values. This is because the same lot was assumed for the as-cracked and the non-damaged specimens, and the same probability density functions were set for the microstructure. In addition, as an example, the result obtained by the reloading, which corresponds to the result in Figure 10, is shown in the black square. Even if the non-damaged specimen is completely healed, the Weibull distribution is considered to slightly shift to the higher strength side under the same boundary conditions. It should be noted that the order (A-E) of the bending strengths magnitude after healing is different from that of as-cracked specimens because the microstructure data around the healed part in the crack-healing material are stochastically distributed.

In this analysis, the scatter of the as-cracked specimen was relatively large. This is because the plane strain condition of one element (z-direction) is adopted, and the bending strength depends on the fracture stress of the element just above the initial damaged part corresponding to the stress concentration area. In the case of ordinary three-dimensional analysis in which many elements are discretized in the z-direction, the scatter of bending strength in the as-cracked specimen is considered to become small.

### 5.3 Comparison with experimental results

**Figure 14.**
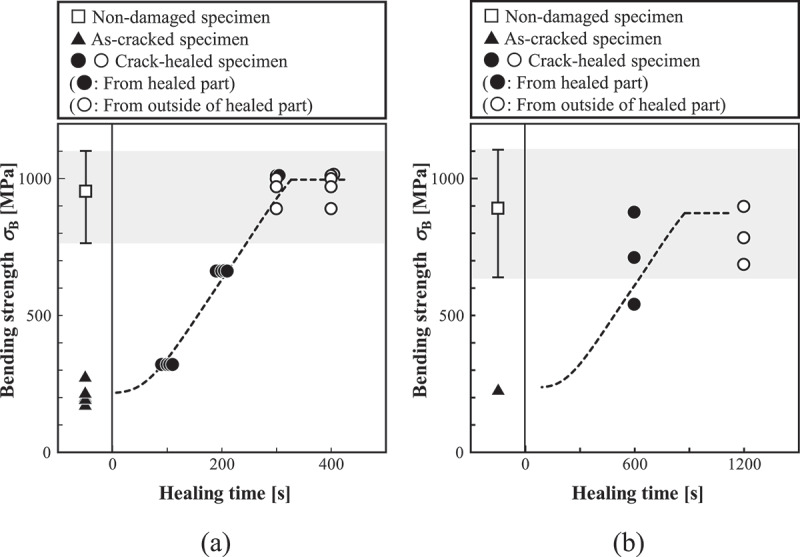
Time dependence of strength recovery obtained by (a) FEA and (b) experiment. The plots correspond to peak values of the bending stress-deflection relationships.

Finally, a qualitative comparison between the FEA results and the experimental results [15] is described. Figure 14(a) and 14(b) show the results of FEA and experiment on the time dependence of strength recovery, respectively. Here, separate plots of the graph show non-damaged, as-cracked, and crack-healed specimens. The empty circles (i.e., ○) in the crack-healed specimen correspond to the cases in which the damage occurred in the parts other than the healed part, and the filled circles (i.e., ●) correspond to the cases occurring in the healed part. Further, the error bar of the non-damaged specimen shows the range between the maximum and minimum values of the Weibull distribution in Figure 13. As shown in the figure, the FEA results demonstrate that the strength of the as-cracked specimen recovered as the healing time increased, and reached the same level as the non-damaged specimen, as in the case of the experiment. It is also confirmed that the strength of the crack-healed specimen varies within the range of the non-damaged specimen scatter. Furthermore, when the healing time is sufficiently long, the strength of the healed part increases. Therefore, the tendency for crack initiation moving around the healed part is also the same.

However, in the experiment, the scatter of strength is observed even when a crack develops from the healed part after healing to some extent. This difference is because the FEA used the plane strain condition. For a quantitative agreement between the FEA and experiment, 3D model that considers both the discretization in the width direction (z-direction) and the microstructure data of the completely healed part are necessary to be appropriately set. Considering these factors, it would be possible to quantitatively examine the time dependence of strength recovery. Even so, the present FEA methodology can express the basic characteristics of recovery and strength scatter in self-healing ceramics. Thus, the self-healing behavior, which is linked with the stochastic fracture simulation due to microstructure distribution, can be evaluated.

## 6. Conclusions

In this study, we applied the previously proposed damage-healing constitutive model to the FEA of a series of damage and healing processes of Al_2_O_3_/15 vol% SiC. In the FEA, the stochastic distribution of the microstructure, such as relative density, size and aspect ratio of pores, as well as grain size, was also considered. We then virtually performed the 3-point bending test and analysis using a Weibull distribution, to examine both the self-healing effect and scatter of ceramics strength. Thereafter, the time dependence of strength recovery obtained through FEA was qualitatively compared with that obtained by the experiment. It was confirmed that the present FEA methodology can reasonably reproduce the basic characteristics of recovery and scatter of strength in self-healing ceramics. Thus, we conclude that the present FEA methodology could be used for studying the self-healing behavior linked with the simulation of stochastic fracture caused by the microstructure distribution, which is essential to the mechanical and materials design of self-healing ceramics.

The advantages of the present method are summarized as follows:

(1) By utilizing the features of the FEM, the present model can be applied to a member/component of arbitrary shape under arbitrary boundary condition.
(2) The fracture of ceramics is based on the weakest link theory; therefore, the size dependence of ceramic strength can be evaluated reasonably.
(3) Since the evolution law for self-healing based on the oxidation kinetics is adopted, analysis can be performed in the arbitrary atmospheric environment (temperature and oxygen partial pressure).
(4) Other damage models, fracture mechanics models, and kinetics models can be applied as needed.

Regarding (4), however, we focused on the fracture process caused by the tensile cracking due to 3-point bending in this study. The effectiveness of the present FEA for mode II and mode III cracking and compressive fractures must be examined in the near future. Furthermore, we adopted the empirical oxidation kinetics equation for evolution laws. To reflect the actual crack-filling phenomena in the constitutive model, more precise kinetics equations should be applied.
